# Ropivacaine versus levobupivacaine in peripheral nerve block: A PRISMA-compliant meta-analysis of randomized controlled trials

**DOI:** 10.1097/MD.0000000000006551

**Published:** 2017-04-07

**Authors:** Ang Li, Zhijian Wei, Yang Liu, Jiaxiao Shi, Han Ding, Haoshuai Tang, Pengyuan Zheng, Yanzheng Gao, Shiqing Feng

**Affiliations:** a Department of Orthopedics, Tianjin Medical University General Hospital, Heping District, Tianjin; b Department of Orthopaedics, Henan Provincial People's Hospital, Zhengzhou, China.

**Keywords:** levobupivacaine, meta-analysis, randomized controlled trials, ropivacaine

## Abstract

**Background:**

To determine which is more potent in peripheral nerve block between ropivacaine and levobupivacaine.

**Methods:**

A literature search was performed in the EMBASE, Medline, the Cochrane Library, and the Web of Science. The trials that were found were then evaluated for eligibility. The Cochrane Collaboration's Review Manager software was used to perform the meta-analyses.

**Results:**

Twelve studies including 556 patients were included for final analysis. No statistically significant difference was observed between the 2 drugs with respect to onset time of surgical anesthesia, onset time of sensory block, onset time of motor block, duration of motor block, and patients overall satisfaction. Levobupivacaine provided more long-term anesthesia (weighted mean difference [WMD], −2.94; 95% confidence interval [CI], −5.56 to −0.32; *I*^2^ = 93%) and significantly lower incidence of postoperative rescue analgesia (odds ratio [OR], 2.11; 95% CI 1.18–3.74; *I*^2^ = 21%) than ropivacaine. There was a trend toward greater duration of sensory block in the levobupivacaine group (WMD, −1.16; 95% CI, −1.89 to −0.43; *I*^2^ = 14%).

**Conclusion:**

Levobupivacaine is more potent than ropivacaine in peripheral nerve block to some extent. Otherwise, more rigorous randomized control trials are required in the future.

## Abstract 2

Supplemental Digital Content is available in the text

## 1 Introduction

Peripheral nerve block, as regional anesthesia, is frequently used for extremity surgeries to optimize severe intraoperative and postoperative pain relief.^[1]^ It can provide sympathetic block, dose-sparing effects of opioids, better perioperative analgesia and offer many advantages over general anesthesia, such as avoidance of respiratory tract administration, reduction of recovery time, and economic cost, and improved patient satisfaction.^[2,3]^ Since the introduction of long-acting local anesthetics (LAs) with better safety clinical profiles, the peripheral nerve block has increased in decade years. Despite its long-acting analgesic properties, concerns about racemic bupivacaine have been raised over its potential cardiotoxicity and central nervous system (CNS) toxicity after inadvertent intravenous administration which may be fatal sometimes.^[4,5]^ To reduce risk of specific toxic characteristics, nonracemic LAs such as ropivacaine or levobupivacaine emerged at the right moment, both of which are the pure left-isomers of bupivacaine and quite similar in physicochemical properties. Both these 2 long-acting LA amides are associated with lower cardiac and CNS toxicity than racemic bupivacaine, having been developed to offer a safer alternative to bupivacaine. Although levobupivacaine is theoretically more potent than ropivacaine, clinical studies show conflicting results in terms of anesthetic and analgesic characteristics. Many studies have showed that equipotent doses of ropivacaine and levobupivacaine have similar efficacy in peripheral nerve plexus block and epidural anesthesia in ambulatory patients, as well as when administered by topical application or local infiltration.^[6,7]^ So, further critical evidence is needed to complete clinical application guidance.

## 2 Methods

### 2.1 Inclusion and exclusion criteria

PRISMA guidelines were followed for the inclusion of studies^[8]^ in the meta-analysis. The detailed description of inclusion criteria are as follows^[1]^: trials had to be properly randomized^[2]^; no additional agents or interventions confounded the comparison (ie, 2 groups had to differ only by addition of either ropivacaine or levobupivacaine)^[3]^; patients were given a bolus dose of LAs^[4]^; and with respect to trials with several intervention groups, the eligibility of each individual group was evaluated and only those qualified were included. However, studies failed to provide enough essential information about the outcomes assessments were excluded. Trials had to focus on peripheral nerve blocks, which aimed to spinal anesthesia involving epidural and subarachnoid anesthesia were also excluded. When patients were blocked only by a continuous infusion, studies were excluded. In addition, when each individual group examining one anesthetic plus another one, or more, (eg, ropivacaine combined with levobupivacaine) the studies were not permitted definitely. Early studies published as a series of articles from the same institution or author that contained significant overlapping data were excluded for fear of multiple publication bias.

### 2.2 Literature search

Both published and unpublished literatures were searched in case of publication bias. The following electronic databases were extensively searched from their inception through August 2016 independently by 2 investigators: EMBASE, Medline, the Cochrane Library, and the Web of Science with keywords centered on the terms “ropivacaine,” “levoupivacaine,” and “peripheral nerve,” which were adjusted to each database in necessity. Besides, bibliographies of the included studies and dissertations were searched for additional publications. The search language was restricted to English.

### 2.3 Data collection

After removing duplicates, titles and abstracts were scanned by 2 independent investigators according to predefined selection criteria and potentially relevant RCTs were selected. Hard copies of all relevant articles were retrieved and read in full for further identification. The relevant data were extracted by adapting a predetermined standardized procedure, which involved first authors, year of publication, country, participants demographic characteristics, and treatment regime for each group. Disagreements regarding studies to be included and data abstraction were resolved by consensus or discussion with a 3rd author.

### 2.4 Quality assessment

Methodological quality assessment of eligible studies were conducted by using the Cochrane risk-of-bias algorithm. The following 7 characteristics were assessed: random sequence generation, allocation concealment, blinding of participants, personnel and outcome assessment, incomplete outcome measures, selective outcome reporting, and other bias. Each item was classified to “low (L), unclear (U), and high (H)” as having a low, unclear, and high risk of bias, respectively. Two independent practitioners met and reviewed every entry for accuracy and consistency, and discrepancies were resolved by consensus.

### 2.5 Statistical analysis

The Cochrane Collaboration Review Manager Software Package (RevMan Version 5.2) was used to perform meta-analyses. The overall effect size of each anesthetic was calculated as weighted average of the inverse variance for study-specific estimates. For dichotomous variables, odds ratios (ORs) with the corresponding 95% confidence interval (CI) were calculated, and correspondingly weighted mean difference (WMD) was used to estimate numerical variables. Heterogeneity was evaluated with the χ^2^ distribution test and Higgins *I*^2^ index, and considerable heterogeneity was determined when the Cochrane Q test resulted in *P* < 0.10 and *I*^2^ above 75%. In such cases, a random effect model was selected for analysis. Conversely, a fixed effect model was used. If essential, subgroup analysis was conducted to identify and explain the heterogeneity, stratified by either anesthetics dose or anesthetics concentration or the kinds of peripheral nerves blocked. When the median, the minimum, the maximum or the 25th and 75th centiles were only available, some methods were used to estimate the sample mean and standard deviation.^[9]^

### 2.6 Outcomes

Eight aspects were assessed to compare the potency of ropivacaine with levobupivacaine in peripheral nerve block for pain management in patients: onset time of surgical anesthesia, onset time of sensory block, onset time of motor block percent patients that need postoperative rescue analgesia, patients overall degree of satisfaction with block, duration of nerve block, duration of sensory block, and duration of motor block.

### 2.7 Ethical statement

As all analyses were grounded on previously published studies, ethical approval was not necessary.

## 3 Results

### 3.1 Characteristics of the trials

**Figure 1:**
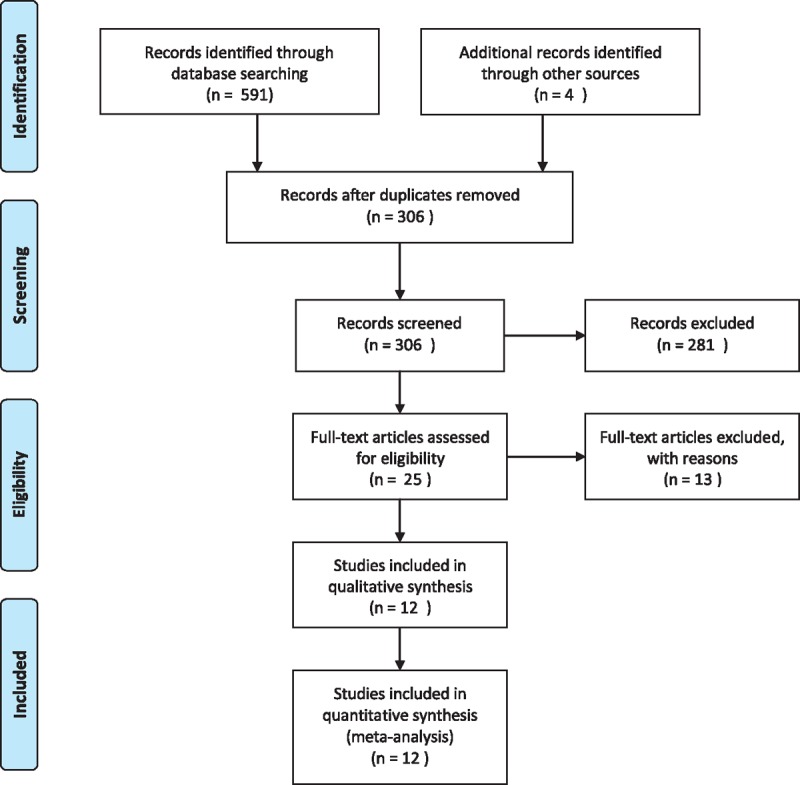
Flow diagram of study selection.

Figure 1 presents a flowchart describing the process by which we screened and selected trials. The initial literature search yielded 595 articles in all. According to inclusion and exclusion criteria, duplicates checking and title and abstract screening resulted in 25 publications. Consequently, 12 articles were analyzed in the meta-analysis. All patients included among studies received a bolus dose of LAs, and the patients also received a continuous infusion after surgery in 1 study.^[10]^ Besides, patients in 1 study received a bilateral selective ankle block: 1 foot was blocked with levobupivacaine and the other with ropivacaine.^[11]^ There were no differences between groups concerning demographic and clinical data in each study. The mean age of the participants ranged between 27 and 71 years. The sample size among trials was with little range from 28 to 86. The detailed characteristics of the eligible trials are shown in the Table 1.

**Table 1:**
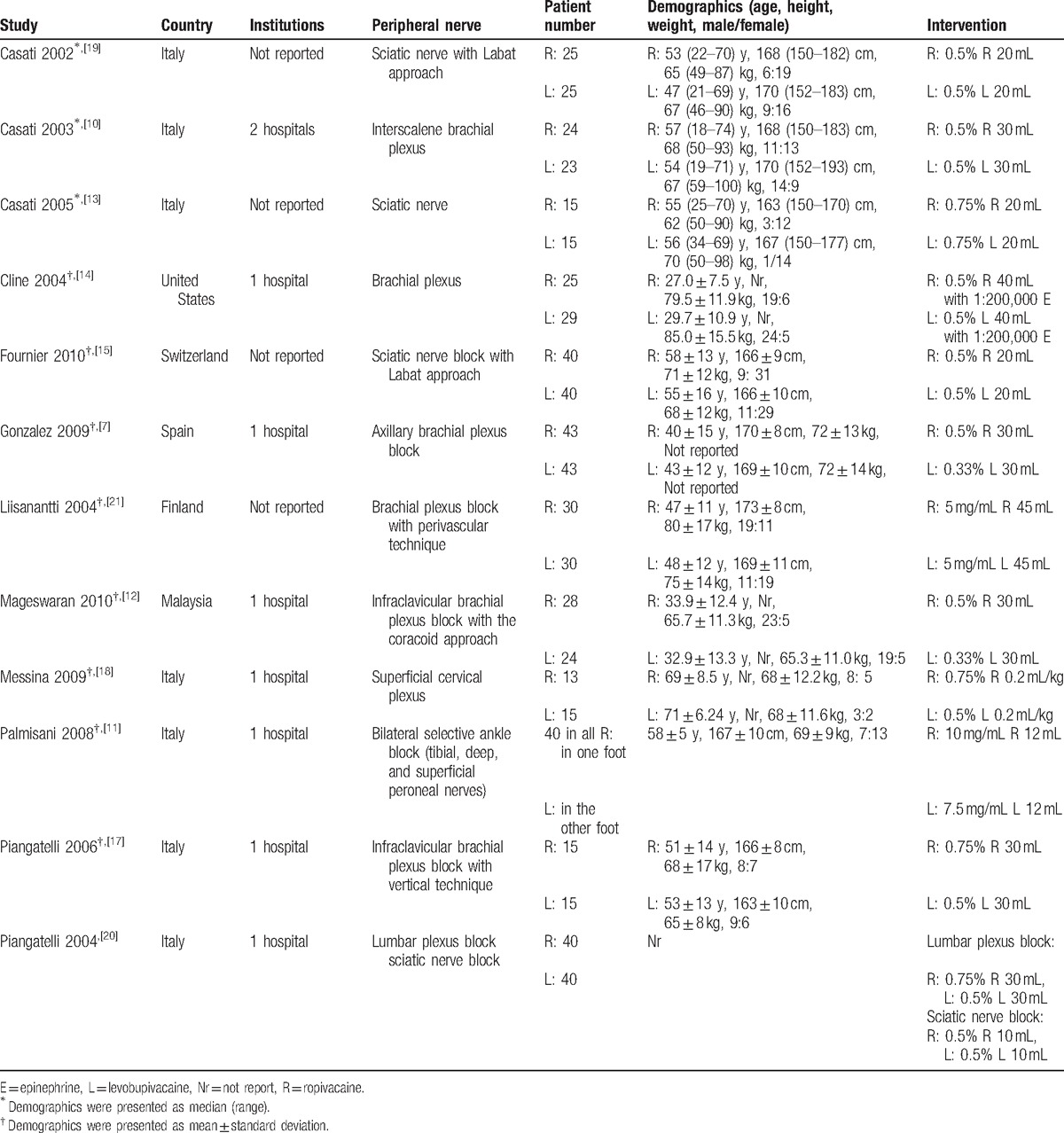
Summary of characteristics in studies included.

The methodological quality of the included trials is summarized in Table 2. The whole 12 studies were typical randomized control trials, with 7 studies^[7,10,11,13–15,18]^ reporting acceptable methods of randomization. All studies, apart from 2 trials conducted by Piangatelli, reported the blinding of both participants and personnel. Additionally, for unexpected drop-outs in process, only 1 study used the intention-to-treat approach in data handling.^[18]^ Whether enrollment of participants was actually consecutive or not was unclear in most of studies, so the attrition and selection bias were unavoidable.

**Table 2:**
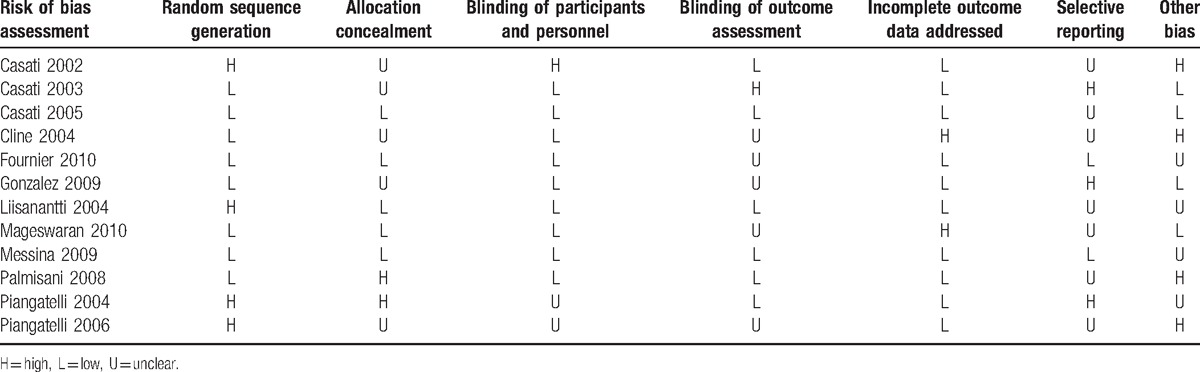
Risk of bias assessment of randomized controlled trials.

### 3.2 Onset time of surgical anesthesia

**Figure 2:**
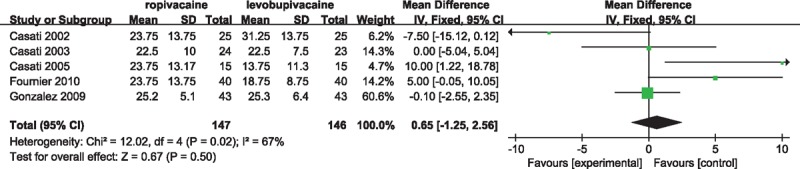
Forest plot for onset time of surgical anesthesia.

Surgical anesthesia was defined as adequate loss of pinprick sensation in nerves distribution and concomitant inability to move the extremities. Five studies^[7,10,13,15,19]^ compared onset time of surgical anesthesia in this meta-analysis. However, no significant differences were observed among studies (WMD 0.65; 95% CI: −1.25–2.56; heterogeneity: χ^2^ = 12.02, *P* = 0.02, *I*^2^ = 67%) (Fig. 2). Onset time of sensory block was reported in 6 trials. No significant difference in the beginning time of adequate sensory block between ropivacaine and levobupivacaine among studies included was observed (WMD −3.57; 95% CI: −8.11–0.98) (Supplemental Fig. 1). The pinprick test was applied to evaluate onset time of sensory block in 5 studies.^[7,11,12,14,17,18]^ Five studies recorded the onset time of motor block as an endpoint, 4 of which was assessed by the Bromage scale.^[12,14,17,20]^ Similarly, no more superiority of levobupivacaine than ropivacaine in term of motor block happened among these studies (WMD 2.01; 95% CI: −1.68–5.70) (Supplemental Fig. 2). However, severe heterogeneity among the studies existed, with the *I*^2^ value be 97% or 94% in sensory or motor block separately. So, random effect models were applied in both 2 measure parameters.

### 3.3 Duration of block

**Figure 3:**
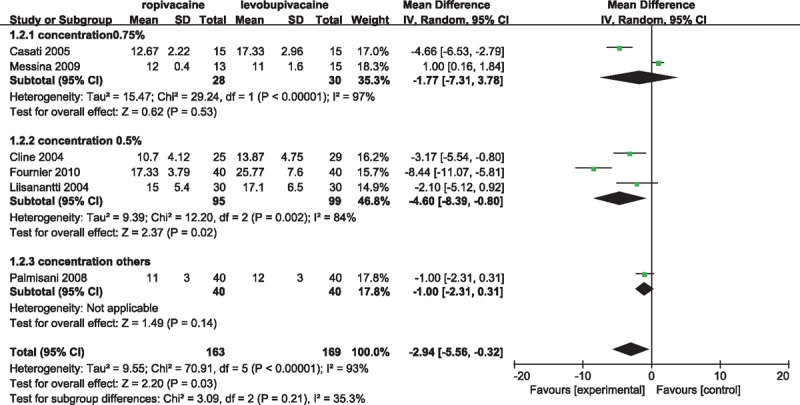
Forest plot for duration of block.

Overall 6 studies^[11,13–15,18,21]^ reported the duration of block. The meta-analysis revealed that levobupivacaine provided longer-term anesthesia than ropivacaine, with a pooled WMD of −2.94 (95% CI −5.56 to −0.32) (Fig. 3). With respect to large statistical heterogeneity that *I*^2^ value was 93%, subgroup analyses were performed to assess the interstudy dose concentration deviation. In the subgroup of concentrations of 0.75%, the results did not differ significantly between 2 drugs. Although concentrations was 0.5%, the duration of block favored the levobupivacaine, similar to the overall pooled effect size. Besides, 3 studies^[7,17,20]^ reported the duration of both sensory and motor block among 6 studies included. There was a trend toward greater duration of sensory block in the levobupivacaine group (WMD, −1.16; 95% CI −1.89 to −0.43; *P* = 0.002; heterogeneity: χ^2^ = 2.32, *P* = 0.31, *I*^2^ = 14%) (Supplemental Fig. 3), while average duration of motor block occurred without any clinically significant differences (WMD, 0.09; 95% CI −0.51–0.69; *P* = 0.76; heterogeneity: χ^2^ = 0.08, *P* = 0.96, *I*^2^ = 0%) (Supplemental Fig. 4).

### 3.4 Postoperative rescue analgesia

**Figure 4:**
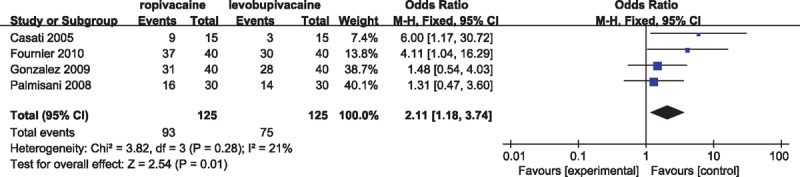
Forest plot for postoperative rescue analgesia.

Four studies^[7,11,13,15]^ compared percentage or number of patients who needed postoperative rescue analgesia. The OR-based models revealed that incidence of postoperative rescue analgesia was significantly higher in ropivacaine group than that in levobupivacaine group (OR, 2.11; 95% CI 1.18–3.74; *P* = 0.01; heterogeneity: χ^2^ = 3.82, *P* = 0.28, *I*^2^ = 21%) (Fig. 4). Except for routine pain medication irrespective of pain status, drugs used for postoperative supplementary analgesia among studies were as following: tramadol 100 mg, subcutaneous morphine 0.1 mg/kg, intravenous metamizol (2 g), and ketorolac 30 mg. Rescue analgesic was used when visual analogue scale was above 30 mm in 3 studies,^[7,13,15]^ while 40 mm in 1 study.^[11]^

### 3.5 Patients overall satisfaction

**Figure 5:**
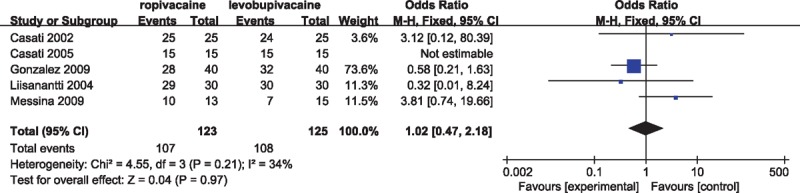
Forest plot for patients overall satisfaction.

The quality or acceptance of the anesthetic technique with either levobupivacaine or ropivacaine was assessed by inclusive patients with certain subjectivity in 5 studies.^[7,13,18,19,21]^ It was noteworthy that there were not some distinct differences in patients overall degree of satisfaction between 2 groups (OR, 1.02; 95% CI 0.47–2.18; *P* = 0.97; heterogeneity: χ^2^ = 4.55, *P* = 0.21, *I*^2^ = 34%) (Fig. 5), and most patients indicated a willingness to accept the same anesthesia procedure for the future operations.

## 4 Discussion

In all, the presented study provides valuable information about the clinical profile of ropivacaine versus levobupivacaine for peripheral nerve blocks in the first time. No statistically significant difference was observed between the 2 drugs with respect to onset time of surgical anesthesia, onset time of sensory block, onset time of motor block, duration of motor block, and patients overall satisfaction. Otherwise, significantly longer duration of block and the sole sensory block in levobupivacaine group existed than those in ropivacaine group. Percent patients who needed postoperative rescue analgesia was significantly lower in levobupivacaine group, compared with that in ropivacaine group.

Identical volumes and concentrations of levobupivacaine and ropivacaine were used to induce the block in the studies included, except one.^[18]^ For pharmacological properties, ropivacaine is about 10 times less lipophilic than levobupivacaine and is therefore resistant to rapidly penetrating the myelinated nerve fibers and easily induces local vasoconstriction in tissues surrounding the injection site.^[16,22]^ Consequently, it might have hindered diffusion of ropivacaine solution within the soft tissues and fat, leaving a high level of concentration solution near the nerves to block. Illustrated by some literatures, adipose tissue can influence regional anesthesia, especially the perineural and epineural fat,^[23]^ leading to a delayed onset time of motor and sensory block and a diminished degree of anesthesia intensity.^[23]^ Multiple studies have reported that ropivacaine possessed a lower potency (of up to 40%), compared with bupivacaine.^[24]^

When comparing the 2 drugs, reported by Fournier et al,^[15]^ the differences must be considered in molarity due to apparent differences in molecular weight and presentation as a hydrochloride salt or a base. It was documented that 225 mg ropivacaine was considered equipotent to 150 mg levobupivacaine.^[25]^ In patient-controlled continuous interscalene analgesia, Borghi et al^[26]^ reported that 0.25% levobupivacaine provided similar quality of anesthesia as the one produced by equipotent (0.4%) concentration of ropivacaine, but better anesthesia than that with equivalent (0.25%) concentration in a similar clinical setting. It was showed that the onset and duration of nerve block induced by equimolar doses of 2 LAs were similar on isolated nerves.^[27]^ So that under this consideration, ropivacaine might be equipotent to levobupivacaine, but more additional factors should be taken into account because of complexity and instability in clinical practice.

Regarding the duration of block, the basic researches indicated that both levobupivacaine and racemic bupivacaine were nearly 50% more effective than ropivacaine in suppressing tetrodoxin-resistant sodium ion channels, which confirmed by some animal experiments.^[28]^ MLAC are estimates of the minimum concentration that provides sufficient anesthesia in 50% of patients.^[29]^ The MLAC was 0.083% (levobupivacaine) and 0.081% (bupivacaine) separately, with approximately 50% higher for ropivacaine, which was in accord with the results of this meta-analysis to some extent. The reason for apparent higher potency of levobupivacaine could be that ropivacaine concentrations were presented as the hydrochloride salt, rather than base like levobupivacaine, which underestimated the concentration by 13%.^[30]^

It was reported that the duration of block was bound up with protein-bound level, and more highly protein-bound drugs could lead a longer duration of effect.^[31]^ Percent protein binding differed slightly but not significantly (94% in ropivacaine vs 95% in levobupivacaine).^[32]^ Besides, the difference in clinical factors such as block technique and magnitude of operations is correlated to duration of analgesia, as Cline et al inferred.^[14]^ Messina et al^[15]^ reported that the poor effect of levobupivacaine could be explained by the choice of a low concentration for this kind of surgery, which was sufficient for analgesia but not for anesthesia. Additionally, Mageswaran and Choy^[12]^ reported that the supraclavicular approach could offer denser anesthesia in brachial plexus block, compared with the infraclavicular block.

It was reported that ropivacaine had a more selective impact on nociceptive (Aδ and C fibers) than on motor fibers, which might give rise to a faster onset of sensory block for ropivacaine.^[33]^ Nevertheless, the meta-analysis revealed that onset time of block, no matter sensation or motor, was significantly equivalent between 2 anesthetics. Though extensive distribution in tissues, the rate of absorption varied largely, which depended on the regional vascularity density and the method of administration. It cannot be suggested that equivalent doses of LAs will produce equivalent effects, which were presented by different or even opposite results in studies included in this meta-analysis.

Overall 8 studies mentioned complications during perianesthesia. Otherwise, only 2 studies reported that relevant complications really existed.^[7,10]^ The primary adverse events noted were nausea and vomiting other than 1 episode of intraoperative bradycardia. Slightly but not significantly more complications occurred in ropivacaine group than levobupivacaine group. It is well known that nausea, hypotension, and anemia are the most common adverse reactions (all at a frequency of ≧10%). However, these adverse events are not necessarily caused by LAs, as surgical procedures or some underlying conditions may answer for these reactions. When the CNS and cardiovascular effects were compared between 2 drugs at equal conditions, no differences in mean percentage changes were reported for relevant parameters such as stroke index, cardiac index, PR interval, and convulsive threshold dose.^[34,35]^ However, it turned out that significantly less adrenaline (epinephrine) was required to treat ropivacaine-induced cardiac arrest than for levobupivacaine-treated rats.^[36]^ In all, both LAs were well tolerated in clinical practice generally.

The small overall patient population and the inconsistencies in some parametric data are important limitations of the meta-analysis. Owing to few data available and confounding factors among studies, the percent of successful blocks, supplementary anesthesia consumption, and postoperative pain intensity were not assessed. Besides, owing to small, single-center trials per treatment group, a center effect was inevitable, which could be excluded by comparing the cases enrolled in the 2 or more participating institutions. Additionally, absolute surgical anesthesia should involve absence of both sensation and motor function of extremities. However, Fournier et al^[15]^ reported that the block was considered successful when just the sensory block was achieved. Measurement standards used and trial methods were not always directly comparable across trials. With respect to duration of block, although subgroup analysis stratified by anesthetics concentration was conducted, substantial heterogeneity could not be prevented, which might reduced reliability of results. Furthermore, the anesthetics were administrated by a bolus dose in studies included in the presented meta-analysis, but continuous infusion model was usually applied in many trials.^[26,37]^

## 5 Conclusions

Administration with levobupivacaine for peripheral nerve block led to significantly longer duration of block, duration of sensory block, and less patients that need postoperative rescue analgesia when compared with those of ropivacaine. However, properly powered studies with a much larger sample size are advocated in order to get a more concrete conclusion.

## Acknowledgements

The authors thank State Key Program of National Natural Science Foundation of China (81330042), Special Program for Sino-Russian Joint Research Sponsored by the Ministry of Science and Technology, China (2014DFR31210), International Cooperation Program of National Natural Science Foundation of China (81620108018), Key Program Sponsored by the Tianjin Science and Technology Committee, China (13RCGFSY19000, 14ZCZDSY00044), and Henan Provincial Innovation and Outstanding Talent Program (154200510027) for the support.

## Supplementary Material

Supplemental Digital Content

## References

[1] Needoff M Radford P Costigan P Local anesthesia for postoperative pain relief after foot surgery: a prospective clinical trial. Foot Ankle Int 1995;16:11–3. 7697147 10.1177/107110079501600103

[2] Salinas FV Joseph RS Peripheral nerve blocks for ambulatory surgery. Anesthesiol Clin 2014;32:341–55. 24882122 10.1016/j.anclin.2014.02.005

[3] Roussel J Thirkannad S Comparison of 3 ultrasound-guided brachial plexus block approaches for cubital tunnel release surgery in 120 ambulatory patients. AANA J 2014;82:121–6. 24902454

[4] Albright GA Cardiac arrest following regional anesthesia with etidocaine or bupivacaine. Anesthesiology 1979;51:285–7. 484889 10.1097/00000542-197910000-00001

[5] Groban L Deal DD Vernon JC Cardiac resuscitation after incremental overdosage with lidocaine, bupivacaine, levobupivacaine, and ropivacaine in anesthetized dogs. Anesth Analg 2001;92:37–43. 11133597 10.1097/00000539-200101000-00008

[6] Taspinar V Sahin A Donmez NF Low-dose ropivacaine or levobupivacaine walking spinal anesthesia in ambulatory inguinal herniorrhaphy. J Anesth 2011;25:219–24. 21225292 10.1007/s00540-010-1089-9

[7] Gonzalez-Suarez S Pacheco M Roige J Comparative study of ropivacaine 0.5% and levobupivacaine 0.33% in axillary brachial plexus block. Reg Anesth Pain Med 2009;34:414–9. 19920417 10.1097/AAP.0b013e3181ae729b

[8] Liberati A Altman DG Tetzlaff J The PRISMA statement for reporting systematic reviews and meta-analyses of studies that evaluate healthcare interventions: explanation and elaboration. BMJ 2009;339:b2700. 19622552 10.1136/bmj.b2700 PMC2714672

[9] Wan X Wang W Liu J Estimating the sample mean and standard deviation from the sample size, median, range and/or interquartile range. BMC Med Res Methodol 2014;14:135. 25524443 10.1186/1471-2288-14-135 PMC4383202

[10] Casati A Borghi B Fanelli G Interscalene brachial plexus anesthesia and analgesia for open shoulder surgery: a randomized, double-blinded comparison between levobupivacaine and ropivacaine. Anesth Analg 2003;96:253–9. 12505962 10.1097/00000539-200301000-00051

[11] Palmisani S Arcioni R Di Benedetto P Ropivacaine and levobupivacaine for bilateral selective ankle block in patients undergoing hallux valgus repair. Acta Anaesthesiol Scand 2008;52:841–4. 18477086 10.1111/j.1399-6576.2008.01630.x

[12] Mageswaran R Choy YC Comparison of 0.5% ropivacaine and 0.5% levobupivacaine for infraclavicular brachial plexus block. Med J Malaysia 2010;65:300–3. 21901950

[13] Casati A Vinciguerra F Santorsola R Sciatic nerve block with 0.5% levobupivacaine, 0.75% levobupivacaine or 0.75% ropivacaine: a double-blind, randomized comparison. Eur J Anaesthesiol 2005;22:452–6. 15991509 10.1017/s0265021505000773

[14] Cline E Franz D Polley RD Analgesia and effectiveness of levobupivacaine compared with ropivacaine in patients undergoing an axillary brachial plexus block. AANA J 2004;72:339–45. 15529729

[15] Fournier R Faust A Chassot O Levobupivacaine 0.5% provides longer analgesia after sciatic nerve block using the Labat approach than the same dose of ropivacaine in foot and ankle surgery. Anesth Analg 2010;110:1486–9. 20304982 10.1213/ANE.0b013e3181d3e80b

[16] Bouaziz H Iohom G Estebe JP Effects of levobupivacaine and ropivacaine on rat sciatic nerve blood flow. Br J Anaesth 2005;95:696–700. 16183680 10.1093/bja/aei242

[17] Piangatelli C De Angelis C Pecora L Levobupivacaine and ropivacaine in the infraclavicular brachial plexus block. Minerva Anestesiol 2006;72:217–21. 16570033

[18] Messina M Magrin S Bignami E Prospective randomized, blind comparison of ropivacaine and levobupivacaine for superficial plexus anesthesia in carotid endoarterectomy. Minerva Anestesiol 2009;75:7–12. 19172143

[19] Casati A Borghi B Fanelli G A double-blinded, randomized comparison of either 0.5% levobupivacaine or 0.5% ropivacaine for sciatic nerve block. Anesth Analg 2002;94:987–90. table of contents. 11916809 10.1097/00000539-200204000-00039

[20] Piangatelli C De Angelis C Pecora L Levobupivacaine versus ropivacaine in psoas compartment block and sciatic nerve block in orthopedic surgery of the lower extremity. Minerva Anestesiol 2004;70:801–7. 15702061

[21] Liisanantti O Luukkonen J Rosenberg PH High-dose bupivacaine, levobupivacaine and ropivacaine in axillary brachial plexus block. Acta Anaesthesiol Scand 2004;48:601–6. 15101856 10.1111/j.0001-5172.2004.00393.x

[22] Casati A Putzu M Bupivacaine levobupivacaine and ropivacaine: are they clinically different? Best Pract Res Clin Anaesthesiol 2005;19:247–68. 15966496 10.1016/j.bpa.2004.12.003

[23] Higuchi H Adachi Y Kazama T Factors affecting the spread and duration of epidural anesthesia with ropivacaine. Anesthesiology 2004;101:451–60. 15277929 10.1097/00000542-200408000-00027

[24] Beilin Y Halpern S Focused review: ropivacaine versus bupivacaine for epidural labor analgesia. Anesth Analg 2010;111:482–7. 20529986 10.1213/ANE.0b013e3181e3a08e

[25] Connolly C Coventry DM Wildsmith JA Double-blind comparison of ropivacaine 7.5 mg ml(-1) with bupivacaine 5 mg ml(-1) for sciatic nerve block. Br J Anaesth 2001;86:674–7. 11575344 10.1093/bja/86.5.674

[26] Borghi B Facchini F Agnoletti V Pain relief and motor function during continuous interscalene analgesia after open shoulder surgery: a prospective, randomized, double-blind comparison between levobupivacaine 0.25%, and ropivacaine 0.25% or 0.4%. Eur J Anaesthesiol 2006;23:1005–9. 16824239 10.1017/S0265021506000962

[27] Dyhre H Lang M Wallin R The duration of action of bupivacaine, levobupivacaine, ropivacaine and pethidine in peripheral nerve block in the rat. Acta Anaesthesiol Scand 1997;41:1346–52. 9422304 10.1111/j.1399-6576.1997.tb04656.x

[28] Brau ME Branitzki P Olschewski A Block of neuronal tetrodotoxin-resistant Na+ currents by stereoisomers of piperidine local anesthetics. Anesth Analg 2000;91:1499–505. 11094008 10.1097/00000539-200012000-00038

[29] Van de Velde M Dreelinck R Dubois J Determination of the full dose-response relation of intrathecal bupivacaine, levobupivacaine, and ropivacaine, combined with sufentanil, for labor analgesia. Anesthesiology 2007;106:149–56. 17197857 10.1097/00000542-200701000-00024

[30] Sanford M Keating GM Levobupivacaine: a review of its use in regional anaesthesia and pain management. Drugs 2010;70:761–91. 20394458 10.2165/11203250-000000000-00000

[31] Buckenmaier CC 3rd Bleckner LL Anaesthetic agents for advanced regional anaesthesia: a North American perspective. Drugs 2005;65:745–59. 15819588 10.2165/00003495-200565060-00003

[32] Leone S Di Cianni S Casati A Pharmacology, toxicology, and clinical use of new long acting local anesthetics, ropivacaine and levobupivacaine. Acta Biomed 2008;79:92–105. 18788503

[33] Bader AM Datta S Flanagan H Comparison of bupivacaine- and ropivacaine-induced conduction blockade in the isolated rabbit vagus nerve. Anesth Analg 1989;68:724–7. 2735537

[34] Stewart J Kellett N Castro D The central nervous system and cardiovascular effects of levobupivacaine and ropivacaine in healthy volunteers. Anesth Analg 2003;97:412–6. table of contents. 12873927 10.1213/01.ANE.0000069506.68137.F2

[35] Bardsley H Gristwood R Baker H A comparison of the cardiovascular effects of levobupivacaine and rac-bupivacaine following intravenous administration to healthy volunteers. Br J Clin Pharmacol 1998;46:245–9. 9764965 10.1046/j.1365-2125.1998.00775.x PMC1873676

[36] Ohmura S [Systemic toxicity of local anesthetics and its clinical management]. Masui 2008;57 (Suppl):S119–25. 22462170

[37] Heid F Muller N Piepho T Postoperative analgesic efficacy of peripheral levobupivacaine and ropivacaine: a prospective, randomized double-blind trial in patients after total knee arthroplasty. Anesth Analg 2008;106:1559–61. table of contents. 18420876 10.1213/ane.0b013e318168b493

