# Supplementary material for: Ropivacaine versus levobupivacaine in peripheral nerve block: A PRISMA-compliant meta-analysis of randomized controlled trials
Source: Medicine (Baltimore). 2017 Apr 7;96(14):e6551. doi: 10.1097/MD.0000000000006551 (PMC5411209; doi:10.1097/MD.0000000000006551)
Supplement: Supplemental Digital Content [file medi-96-e6551-s001.doc]

Supplemental Figure 1: Forest plot for onset time of sensory block

Supplemental Figure 2: Forest plot for onset time of motor block

Supplemental Figure 3: Forest plot for duration of sensory block

Supplemental Figure 4: Forest plot for duration of sensory block
